# Clinical anticancer drug development: targeting the cyclin-dependent kinases

**DOI:** 10.1038/sj.bjc.6602229

**Published:** 2004-11-23

**Authors:** C Benson, S Kaye, P Workman, M Garrett, M Walton, J de Bono

**Affiliations:** 1 Section of Medicine and Cancer Research UK Centre for Cancer Therapeutics, Institute for Cancer Research and Royal Marsden Hospital, Downs Road, Sutton, Surrey SM2 5PT, UK

**Keywords:** cell cycle, targeted therapy

## Abstract

Cell division involves a cyclical biochemical process composed of several step-wise reactions that have to occur once per cell cycle. Dysregulation of cell division is a hallmark of all cancers. Genetic and epigenetic mechanisms frequently result in deranged expression and/or activity of cell-cycle proteins including the cyclins, cyclin-dependent kinases (Cdks), Cdk inhibitors and checkpoint control proteins. The critical nature of these proteins in cell cycling raises hope that targeting them may result in selective cytotoxicity and valuable anticancer activity.

## STRATEGIES FOR TARGETING THE CELL CYCLE

Dysregulation of the cell cycle is a hallmark of malignancy (Table 1). Many different strategies for targeting the cell cycle have been described. Attention has focused primarily on targeting mitosis (by targeting tubulin and more recently mitotic kinases including KSP/Eg5, Polo-like kinase 1 and Aurora kinases) as well as chemical inhibitors of cyclin-dependent kinase (Cdk) catalytic activity. Other potential strategies include therapeutics that can inhibit the interaction between cyclins and Cdks; decrease cyclin expression; promote the degradation of cyclins by increasing their phosphorylation; and restore endogenous Cdk inhibitor function. Other possibilities include the inhibition of the ‘noncycling’ cyclin-activating kinase complex of Cdk7/cyclin H, and the indirect targeting of the late G2 to M checkpoint by inhibiting CDC25 or by activating WEE1 or MYT1. This review will, however, focus on the chemical Cdk inhibitors evaluated in clinical trials to date and will highlight the main trial results reported to date.

## PHARMACOLOGIC CDK INHIBITORS

Considerable progress has been made in the identification of pharmacologic agents targeting the Cdks (Senderowicz, 2003). The first generation of Cdk inhibitors lacked specificity, with flavopiridol, staurosporine and its analogue UCN-01 and E7070 being nonselective inhibitors of not only Cdks but many other targets. Second-generation inhibitors are more selective, with many of these compounds specifically developed to target selected Cdks (Table 2).

### Flavopiridol

Flavopiridol (Alvocidib™) has several mechanisms of anticancer activity (Senderowicz and Sausville, 2000) and is a broad-spectrum Cdk inhibitor targeting Cdks 1, 2, 4, 6 and 7, interacting with the adenosine triphosphate (ATP) binding site. Flavopiridol also inhibits the Cdk9/cyclin T complex, broadly repressing transcription and decreasing cyclin D1 mRNA expression (Carlson *et al*, 1999). It induces cell-cycle arrest in G1 and is cytotoxic to cells undergoing DNA synthesis. It also inhibits other kinases including PKC and PKA at higher concentrations, inducing apoptosis, (Patel *et al*, 1998) and is active in many xenograft models.

The first Phase I trial of flavopiridol utilised a 72-h continuous infusion every 2 weeks, a schedule supported by preclinical models (Senderowicz *et al*, 1998). The maximum-tolerated dose (MTD) achieved in this clinical trial was 50 mg m^2^ day^−1^, the initial dose-limiting toxicity being secretory diarrhoea. The MTD with antidiarrhoeal prophylaxis was 78 mg m^2^ day^−1^ (Kahn *et al*, 2001; Messmann *et al*, 2003) The achieved plasma levels were sufficient for Cdk inhibition (200–400 nM), with one partial response in renal cell cancer and minor responses in renal, colorectal carcinomas and non-Hodgkin's lymphoma.

This 72-h schedule was subsequently investigated in further Phase I and Phase II trials in patients with mantle cell lymphoma, renal cancer, melanoma, gastric cancer, non-small-cell lung cancer (NSCLC) and colorectal cancers (Bennett *et al*, 1999; Stadler *et al*, 2000; Schwartz *et al*, 2001; Shapiro *et al*, 2001; Lin *et al*, 2002; Thomas *et al*, 2002). An unusually high incidence of arterial and venous thromboembolic events was documented in the majority of these trials, including deep venous thrombosis, pulmonary thromboembolism, myocardial infarction and transient neurological ischaemic events. This serious drug-related toxicity was not appreciated in the initial studies but was reported in more than 30% of patients in later trials and is of unknown aetiology.

A study of flavopiridol administered as a 1-h infusion was then pursued supported by data indicating that higher plasma concentrations than those achieved in the 72-h infusion studies were required to induce apoptosis (Arguello *et al*, 1998; Tan *et al*, 2002). Initially, a 1-h infusion administered for 5 days every 3 weeks was investigated. The recommended Phase II dose for this schedule was 37.5 mg m^2^ day^−1^, with grade 4 neutropenia and grade 3 fatigue being dose limiting. The three- and one-day schedules administered every 3 weeks were also evaluated with the aim of further increasing peak serum levels of drug. Grade 4 neutropenia was also dose limiting in these studies, with the recommended Phase II dose of these schedules being 50 mg m^2^ day^−1^ and 62.5 mg m^−2^, respectively. The peak plasma concentrations, however, remained lower than the concentrations required in preclinical studies to induce apoptosis (5–7 *μ*mol l^−1^), with no objective tumour responses being observed. Phase II studies of flavopiridol administered by 1-h infusion daily for 3 days every 3 weeks have been reported. No responses were observed in advanced melanoma (Burdette-Radoux *et al*, 2002); however, in patients with advanced mantle cell lymphoma, three responses out of 28 evaluable patients (11%) were reported, with 20 patients having stable disease (71%) for a median duration of 3.4 months (Kouroukis *et al*, 2003).

An alternative administration schedule has been recently evaluated for flavopiridol in patients with chronic lymphatic leukaemia. Previous Phase I/II studies had shown that, while flavopiridol induces apoptosis in CLL cells in a p53-independent manner *in vitro*, the drug is inactive using a 24- to 72-h CIVI schedule. Based on pharmacokinetic modelling data demonstrating high drug binding to human plasma proteins, an optimised dosing schedule of 30-min i.v. bolus (IVB) followed by 4-h CIVI has been pursued. This alternative schedule has demonstrated promising activity including flavopiridol-induced tumour lysis syndrome, suggesting that this agent may warrant further evaluation using this novel schedule (Lin *et al*, 2004).

The use of flavopiridol in combination studies has also been pursued (Bible and Kaufmann, 1997; Motwani *et al*, 2001). Treatment scheduling appears to be important in ensuring that flavopiridol augments the effects of other agents (Schwartz *et al*, 2002; Wittmann *et al*, 2003).

## UCN-01

The second chemical Cdk inhibitor to be evaluated was UCN-01 (7-hydroxystaurosporine) (Senderowicz and Sausville, 2000), which also has several mechanisms of action including Cdk1 (cdc2) and Cdk2 inhibition (IC_50_ of 300–600 nM). UCN-01 causes cell-cycle arrest and apoptosis at concentrations above 100nM (Akiyama *et al*, 1997), abrogating the G_2_ checkpoint in response to DNA damage and promoting the induction of p53-independent apoptosis by inhibiting Chk1 and Chk2 (IC_50_ for both approximately 10–30 nM). (Wang *et al*, 1996; Busby *et al*, 2000; Yu *et al*, 2002). UCN-01 also abrogates S-phase arrest in CHO cells treated with cisplatin, promoting the induction of apoptosis (Bunch and Eastman, 1997), and may target Akt signalling by inhibiting PDK1 (IC_50_=33 nM) (Sato *et al*, 2002). Frequent dosing is required to optimise the antitumour activity of UCN-01 (Sausville *et al*, 1998), and 72 h of drug exposure is required to achieve growth inhibition. A Phase I trial of UCN-01 administered as a 72-h continuous infusion every 2 weeks to patients with advanced malignancy revealed long elimination half-lives due to high-affinity binding of UCN-01 to *α*1-acid glycoprotein in human plasma (Fuse *et al*, 1998; Sausville *et al*, 2001). This schedule was therefore changed to a 72-h continuous infusion administered every 4 weeks, with a recommended dose of 42.5 mg m^2^ day^−1^. Dose-limiting toxicities at 53 mg m^−2^ included hyperglycaemia, pulmonary toxicity with hypoxaemia, emesis and hypotension. The basis for the pulmonary toxicity was unclear but did not involve altered cardiac motility or pulmonary thromboembolism, being associated with small, transient, pleural effusions. Hyperglycaemia occurred at all dose levels. Analysis of immunoreactive C peptide levels suggested that this was related to peripheral tissue insulin resistance rather than a decrease in insulin secretion. Pharmacokinetic analysis revealed a terminal half-life of 588 h, with mean drug plasma concentrations in the micromolar range. Analysis of free salivary levels of UCN-01 showed drug levels of approximately 100 nm, which is sufficient to affect cell-cycle parameters *in vitro.* A partial response of 6 months duration was observed in patient with melanoma, and a complete response, sustained for over 38 months in a patient with anaplastic large-cell lymphoma.

### E7070

E7070 is a chloroindolyl sulphonamide that induces G1/S cell cycle arrest at low nanomolar concentrations, inhibiting Cdk2/cyclin E, downregulating cyclin H, upregulating p53 and p21, and inducing apoptosis (Ozawa *et al*, 2001). Its potency is enhanced by longer drug exposures (Ozawa *et al*, 2001). E7070 has a broad spectrum of *in vitro* and *in vivo* preclinical antitumour activity, inducing regression of established tumours. This efficacy is schedule dependent, with a daily-for-8-days schedule being more efficacious than 4- and 1-day schedules.

A number of Phase I trials of this agent have been performed investigating different dosing regimens. When given daily by intravenous infusion for 5 days every 3 weeks, the DLTs consisted of neutropenia, thrombocytopenia, diarrhoea and stomatitis, with a recommended Phase II dose of 130 mg m^2^ day^−1^. One partial response was seen in a patient with heavily pretreated breast cancer (Punt *et al*, 2001). A second trial investigated an alternative schedule – a 1-h infusion administered every 3 weeks (Raymond *et al*, 2002). Toxicities were similar to the 5-day regimen and comprised myelosuppression, acne-like skin rash, alopecia, mucositis, conjunctivitis, hypoglycaemia, nausea and fatigue. Recommended doses were 700 mg m^−2^ for the heavily pretreated group and 800 mg m^−2^ for the lightly pretreated group. No partial responses were observed. A weekly 1-h infusion schedule for 4 consecutive weeks and a continuous 120-h infusion have also been evaluated with similar toxicities being observed. The recommended doses for the weekly and continuous infusion schedules were 400 and 96 mg m^−2^, respectively. Pharmacokinetic studies indicate that the clearance and volume of distribution at steady state of E7070 decrease with increasing dose.

Phase II single-agent studies are under way in a number of different tumour types investigating the once-every-3-weeks and daily-for-5-days schedules. In patients with fluorouracil refractory colorectal cancer, two of 21 patients receiving the once-every-4-weeks schedule, and two of 23 patients receiving the daily-for-5-days schedule, had objective responses, with 10 and 13% of patients, respectively, having stable disease at 6 months (Mainwaring *et al*, 2002). In a Phase II study of patients with NSCLC, who had previously received one chemotherapy regimen, only one of 44 patients had an objective response (Talbot *et al*, 2002). No objective responses were observed in patients with metastatic melanoma receiving a dose of 700 mg m^−2^ for over 1 h every 3 weeks (Aamdal *et al*, 2002).

### *R*-Roscovitine (CYC202)

The purine analogue *R-*roscovitine (CYC202) is a highly selective, orally bioavailable, small molecule inhibitor of several Cdks competing with their ATP binding sites: it is a relatively potent inhibitor of human Cdk2/cyclin E, Cdk7/cyclin H, Cdk9/cyclin T1 with IC_50_ of 0.1, 0.5 and 0.8 *μ*M, respectively (Meijer *et al*, 1997), but inhibits Cdk4/cyclin D1 with an IC_50_ of 14.2 *μ*M. It has an average IC_50_ against the NCI cell-line panel of 16 *μ*M. It also blocks the degradation of p53 through the inhibition of MDM2 expression (Lu *et al*, 2001). CYC202 induces G_1_ and G_2_/M arrest and cell death from all compartments of the cell cycle (Schutte *et al*, 1997; McClue *et al*, 2002). The antitumour efficacy of CYC202 has also been tested in a panel of human tumour xenografts (McClue *et al*, 2002). Continuous exposure to CYC202 at dosages ranging from 0.3 to 100 *μ*M demonstrated dose-dependent antitumour activity.

Recent molecular pharmacology studies have shown that treatment of colorectal cancer cells with CYC202 results in a decrease in pRb phosphorylation (serines 780, 608, 807, 811 and Thr-821), indicative of direct Cdk2 inhibition (Whittaker *et al*, 2004). In addition, CYC202 causes a downregulation of various cyclins, including cyclin D1; this is likely to lead to a secondary inhibition of various cyclins, which would explain the reduced phosphorylation at multiple sites on RB that is seen at later time points.

CYC202 has been evaluated in Phase I clinical trials. Two such trials have recently been reported. In the first trial, CYC202 was administered orally, twice daily for 7 days out of every 21. In all, 19 patients were treated for a total of 36 cycles of CYC202. At 800 mg BD, DLTs comprising grade 3 skin rash and grade 4 hypokalaemia were observed. Other toxicities seen included reversible renal impairment, mild reversible transaminitis and emesis. MAG-3 studies indicated that the renal impairment was related to altered renal blood flow. No evidence of renal tubular damage was detected. The aetiology of these renal changes is unknown but could be related to the effects of CYC202 on adenosine receptors. The pharmacokinetics of the compound were dose proportional, with CYC202 being widely distributed (720.8 l, 95% CI 384.9–1056.8) and rapidly cleared (142.6 l/h, 95% CI 80.5–204.9) with a mean terminal elimination half-life of 4.02 h (95% CI 2.8–5.2). This trial is still recruiting patients, but no objective responses have yet been seen (Benson *et al*, 2003; White *et al*, 2004).

In the second trial, a twice-daily-for-5-days schedule, administered every 3 weeks was evaluated. The maximum twice-daily dose achieved was 1600 mg BD with a recommended dose of 1250 mg BD. Toxicities reported have included grade 4 emesis, grade 3 asthenia and skin rash. No objective responses were seen but stable disease was recorded in three out of 29 patients treated. (Pierga *et al*, 2003) Exploration of a 10-day schedule is now under way.

### BMS-387032

High-throughput screening followed by lead optimisation has resulted in the identification of the 2-aminothiazole BMS-387032 as a potent, selective and competitive small molecule inhibitor of the Cdk2/cyclin E complex, with an IC_50_ of 48 nM. The 2-aminothiazoles have been reported to be 10- and 30-fold more potent against Cdk2 than Cdk1 and Cdk4, and three to five orders of magnitude less potent against all other tested non-Cdk kinases. The X-ray crystal structure of this compound with Cdk2 has been reported, and has revealed the mechanisms by which this compound interacts with the Cdk2 ATP binding site (Misra *et al*, 2004). *In vitro*, BMS-387032 inhibits Cdk2 phosphorylation in the A2780 ovarian carcinoma cell line, inhibiting the phosphorylation of downstream targets of Cdk2 including pRb, histone H1 and DNA polymerase-*α*.

The compound displays potent *in vitro* cytotoxicity against the A2780 cell line with an IC_50_ of 50 nM, and is active against a broad array of cell lines. *In vivo* studies have confirmed oral bioavailability and activity against a variety of cell lines, including P388 murine leukaemia, A2780 ovarian and A431 human squamous cell carcinoma. Combination studies indicate that BMS-387032 is synergistic with cisplatin in SV-1 colon carcinoma cells, this synergy being dependent on the drug sequence (Lane *et al*, 2003). Phase I clinical trials with BMS-387032 are ongoing utilising different schedules (Jones *et al*, 2003; McCormick *et al*, 2003; Shapiro *et al*, 2003).

## PHARMACODYNAMIC STUDIES

As with other molecularly targeted therapeutics under investigation, the clinical development of the Cdk inhibitors in early trials requires the study of their biological effects in tumour cells acquired through serial tumour biopsies. These studies are required to select optimal biological dosing and schedule, and identify the patient population most likely to benefit from these agents. Potential pharmacodynamic parameters under investigation include the inhibition of pRb phosphorylation; the depletion of cyclins and Cdks; increases in Cdk inhibitor protein expression; and the suppression of mdm2 expression and induction of p53 expression. Pharmacodynamic studies have been reported for flavopiridol (Lam *et al*, 2001) and CYC202 (Whittaker *et al*, 2004). More recent studies indicate that ^18^F-labelled 3′-deoxy-3′fluorothymidine may be a useful imaging modality for the selective Cdk2 inhibitor BMS-387032 (Fischman *et al*, 2004). These translational studies are critically important in the optimal development of these agents.

## CONCLUDING REMARKS

Significant progress has been made in the clinical targeting of the Cdks. Newer and more specific Cdk inhibitors are envisioned to result in decreased toxicity and more selective cytotoxicity. Increased specificity may not, however, spare noncycling cells since recent data have implicated the Cdks 5, 7, 8 and 9 in cellular functions that do not involve the cell cycle. Cdks 7, 8 and 9 have been reported to regulate RNA transcription through the phosphorylation of RNA polymerase II, while Cdk5 has been shown to be involved in regulating insulin secretion, synaptic vesicle recycling, neuronal survival and tau (microtubule associated protein) phosphorylation and aggregation (Sausville, 2002). These findings may explain why hyperglycaemia has been observed with many chemical Cdk inhibitors. The RNA polymerase II regulatory activity of Cdk 7, 8 and 9 may, however, enhance the antitumour effect of these agents, and it has indeed been suggested that the anticancer activity of flavopiridol may be in part related to the inhibition of RNA transcription. It remains to date unclear whether the inhibition of Cdk 7, 8 and 9 activity is desirable for an anticancer agent, or whether the toxicity associated with inhibiting these noncycling Cdks will substantially decrease cytotoxic selectivity and the therapeutic index.

Overall, these data suggest that highly selective small molecule inhibitors of specific Cdks may be preferable in order to decrease toxicity. Generating this specificity, however, not only remains a significant challenge to chemists but may also decrease anticancer efficacy in view of the inherent functional redundancy of this family of kinases. A broader-spectrum inhibitor that can, for example, selectively inhibit Cdk1, Cdk2, Cdk4 and Cdk6 at low nanomolar IC_50_ concentrations may therefore be preferable. The recent observations that selectively inhibiting Cdk2 in certain cell lines is not sufficient for antitumour activity would support this view (Tetsu and McCormick, 2003), as would the demonstration that the Cdk2 knockout mouse shows no major abnormalities and in particular no effects on proliferation (Ortega *et al*, 2003). The high sequence homology of the ATP binding sites of these and the noncycling kinases Cdk 5, 7, 8 and 9, as well as that of glycogen synthase kinase-3*β* and that of the adenosine receptors makes this a difficult task. However, much has already been achieved and while many questions need to be answered we are moving closer to the Holy Grail: the development of compounds selectively cytotoxic to tumour cells, yet sparing normal cells.

## Figures and Tables

**Table 1:** (a) Deregulated cyclins and Cdk's and associated tumours and (b) deregulated endogenous Cdk inhibitors and associated tumours

**Target**	**Oncogenic changes**	**Associated tumours**
*(a)*		
Cyclin D1	Gene amplification	40–80% Breast carcinoma
	Overexpression	70% Familial polyposis
	Translocation	50% B-cell lymphoma
		50% NSCLC
		35% Head and neck carcinoma
		25–50% Oesophageal carcinoma
		25% Bladder carcinoma
		
Cyclin E	Gene amplification	90% Colorectal carcinoma
	Overexpression	30–80% Breast carcinoma
		70% Prostate carcinoma
		18% Ovarian carcinoma
		Gastric carcinoma
		Cervical carcinoma
		
Cyclin E2	Overexpression	Breast carcinoma
		Small-cell lung carcinoma
		Cervical carcinoma
		
Cyclin B1	Overexpression	90% Colorectal carcinoma
Cyclin A	Amplification or overexpression	Hepatocellular carcinoma
CDK2	Overexpression	Colorectal carcinoma
CDK4	Amplification	Sarcomas, gliomas
		
*(b)*		
p16^INK4a^	Mutation (5% of human cancers)	Pancreatic cancer
	Deletion (14% of human cancers)	Melanoma
	Epigenetic (19% of human cancers)	Gliomas
		Bladder cancer
		Head and neck cancer
		NSCLC
		Lymphoma/leukaemia
		
p21^cip−1/waf−1^	Mutation/deletion rare	Oral (rare mutations)
	Downregulation rare	Oesophageal (rare mutations)
	Intracellular mislocalis ation?	Breast (rare mutations)
		
p27^kip−1^	Mutations/deletions rare	Breast^a^
	Downregulation (increased degradation)^a^	Colon^a^
		Prostate^a^

NSCLC=non-small-cell lung cancer.

a Increased degradation.

**Table 2:** Chemical Cdk inhibitors and their targets

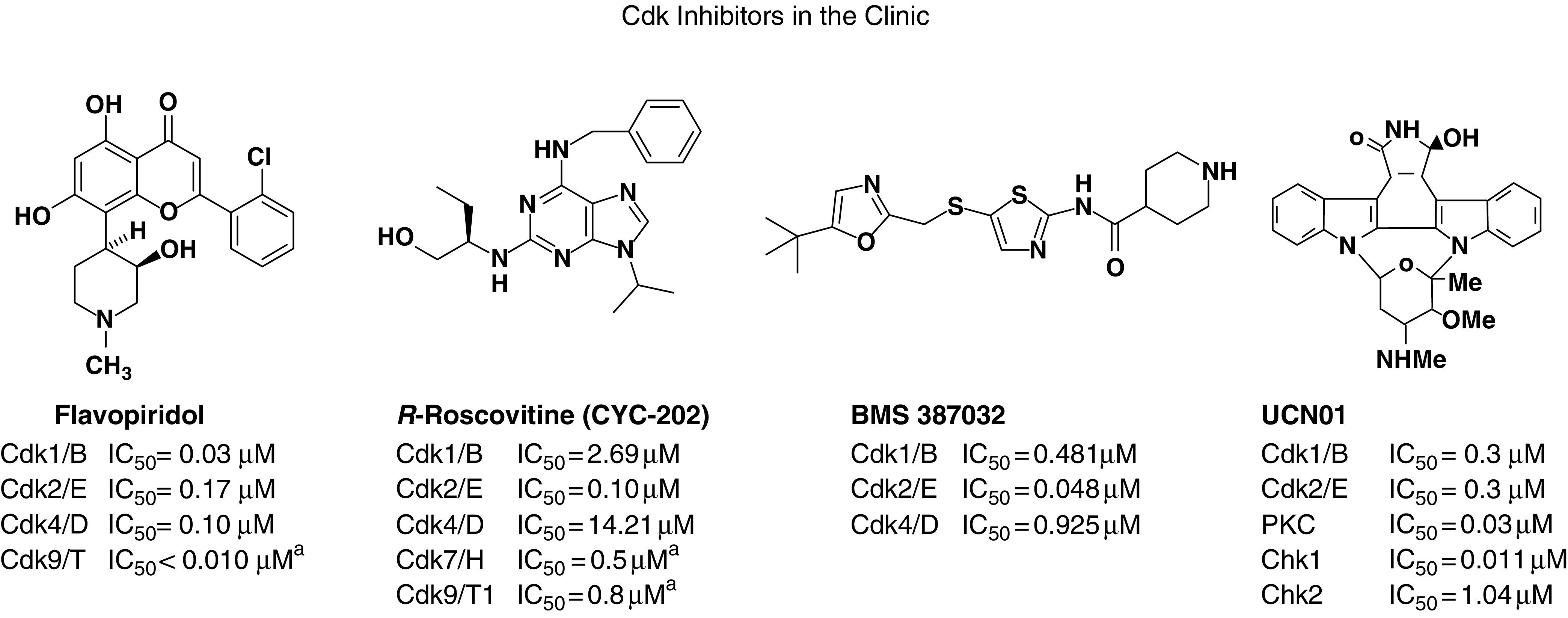

^a^Increased degradation downregulate transcription, for example, ↓cyclin D1 expression.
